# Applicability of Nanopore-only whole-genome sequencing for *Pseudomonas aeruginosa* outbreak investigation in the ICU setting: a multicentric study

**DOI:** 10.1128/jcm.00572-26

**Published:** 2026-07-27

**Authors:** J. Dingemans, S. Vandersanden, P. Hilkens, J. Godelaine, K. Magerman, F. Crombé, D. De Geyter, A. Boel, P. Bruynseels, J. Frans, P. Vandecandelaere, A. M. Van den Abeele, R. Cartuyvels

**Affiliations:** 1 Clinical Laboratory of Molecular Microbiology, Jessa Hospital 37470, Hasselt, Belgium; 2 Faculty of Medicine and Life Sciences, Biomedical Research Institute, Hasselt University 54496, Hasselt, Belgium; 3 Clinical Laboratory of Microbiology, Jessa Hospital 37470, Hasselt, Belgium; 4 Department of Immunology and Infection, Hasselt University 54496, Hasselt, Belgium; 5 Department of Microbiology and Infection Control, Vitality Research Group, Vrije Universiteit Brussel (VUB), Universitair Ziekenhuis Brussel (UZ Brussel) 60201, Brussels, Belgium; 6 Department of Laboratory Medicine, AZORG 694209 , Aalst, Belgium; 7 Department of Laboratory Medicine, Ziekenhuis aan de Stroom 682411, Antwerp, Belgium; 8 Department of Clinical Microbiology, Imelda Hospital 81874, Bonheiden, Belgium; 9 Department of Laboratory Medicine, Jan Yperman Hospital 82282 , Ypres, Belgium; 10 Department of Laboratory Medicine, AZ Sint-Lucas Ghent 82218 , Ghent, Belgium; Children's Hospital Los Angeles, Los Angeles, California, USA

**Keywords:** intensive care unit, outbreak investigation, *Pseudomonas aeruginosa*, Nanopore-only WGS

## Abstract

*Pseudomonas aeruginosa* outbreaks frequently occur in intensive care units (ICUs). In particular, ICU patients requiring mechanical ventilation are vulnerable to *P. aeruginosa* ventilator-associated pneumonia, which is associated with high morbidity and mortality. Fast and accurate genotyping during the early stage is crucial to document and manage *P. aeruginosa* outbreaks at the ICU. In this study, we have evaluated the applicability of Oxford Nanopore whole-genome sequencing (WGS) for outbreak investigation and antimicrobial resistance (AMR) prediction. To evaluate whether a Nanopore-only WGS workflow was able to reproduce Illumina-confirmed transmission clusters, 19 *P. aeruginosa* isolates from ICUs at UZ Brussels (Belgium) that were previously sequenced with Illumina were sequenced using a Nanopore-only workflow based on the latest V14 chemistry, followed by bioinformatic analysis via BugSeq and MBioSEQ Ridom Typer. Although both bioinformatic platforms showed high concordance between Illumina and Nanopore data, MBioSEQ Ridom Typer yielded the lowest allelic distance (maximum one cgMLST allele), confirming all outbreak clusters. When applying the Nanopore-only workflow to longitudinally collected isolates, low genetic heterogeneity (maximum three cgMLST alleles) was observed between isolates from the same patient. WGS and subsequent outbreak analysis of 65 respiratory *P. aeruginosa* isolates collected from 38 different ICU patients across six Belgian hospitals during a 9-month period showed no intra- or inter-hospital transmission. When the Nanopore-only WGS data were used to predict AMR, there was high categorical agreement (95%) between AMR genotype and phenotype. These findings highlight the potential of Nanopore WGS as a rapid and accurate tool for outbreak investigation of *P. aeruginosa*.

**IMPORTANCE:**

In recent years, Nanopore sequencing has found its way to clinical laboratories because of its affordability, scalability, and, most importantly, its ability to obtain sequencing results in near-real time. However, despite improved raw read accuracies with the latest generation R10.4.1 flow cells, the question remains whether the achieved accuracy is sufficient for accurate bacterial outbreak investigation, particularly in high-risk settings such as intensive care units (ICUs). In this study, we show that Nanopore-only whole-genome sequencing (WGS) is able to match Illumina-only WGS in terms of accuracy for *Pseudomonas aeruginosa* outbreak investigation in the ICU setting, although important sequence type-dependent and even strain-specific methylation issues need to be resolved in order to guarantee this accuracy. By providing a fast and accurate workflow for reliable *P. aeruginosa* outbreak investigation, this study could pave the way for large-scale implementation of Nanopore-only WGS, leading to faster outbreak response times.

## INTRODUCTION

Hospital-acquired infections represent a significant challenge for healthcare worldwide, particularly affecting vulnerable populations such as the elderly, neonates, and immunocompromised patients (1). Increasing drug resistance significantly worsens this problem, threatening both individual patient safety and global public health (2). *Pseudomonas aeruginosa* is an important human pathogen causing severe infections (e.g., pneumonia, urinary tract infection, and bloodstream infection), which are associated with significant morbidity and mortality, particularly in patients with risk factors such as immunosuppression, chronic obstructive pulmonary disease (COPD), and cystic fibrosis (3, 4). The emergence of antibiotic-resistant *P. aeruginosa* strains poses a global challenge. Given its significance as a hospital-acquired pathogen, it is essential to understand the transmission dynamics of *P. aeruginosa* to effectively manage and control outbreaks. Illumina short-read (SR) sequencing is considered the gold standard for outbreak investigation. However, recent advancements in Nanopore whole-genome sequencing (WGS), offering a compelling alternative with some unique advantages, have significantly closed the performance gap. Previously, Nanopore sequencing faced challenges with elevated error rates compared to SR methods, particularly affecting its utility in high-resolution genomic analyses. These limitations have been reduced by recent advances in DNA library preparation chemistry (Q20+), flow cells (R10), and basecalling models, resulting in an increased raw read accuracy to around 99%. The latest V14 chemistry upgrade, combined with R10.4 flow cells featuring a double reader-head design, further enhances accuracy, making Nanopore WGS a powerful tool for genomic research (5–7). Nanopore WGS offers faster, real-time, and cost-effective genomic investigations, which are especially valuable during outbreaks. Providing comprehensive genome assemblies comparable to Illumina-derived data but with notably reduced fragmentation, Nanopore sequencing facilitates detailed characterization of bacterial genomes, including the detection of antimicrobial resistance (AMR) genes, plasmids, genes encoding virulence factors, and other genomic elements that may influence the strain’s phenotypic characteristics (5–8). In addition, it offers the advantage of resolving the genomic context of AMR genes, distinguishing between plasmid-encoded and chromosomal resistance (9). Sequencing accuracy can be affected by basecalling errors related to kit chemistry, complex genomic regions, or DNA methylation (10–12). Differences in methylation patterns among species and strains can influence basecalling accuracy (7). As a result, variation in methylation may lead to incorrect allele calls. This study aimed to assess the performance of Oxford Nanopore WGS for outbreak investigation and AMR prediction for *P. aeruginosa* in the intensive care unit (ICU) setting. This was achieved by performing WGS and subsequent outbreak analysis of 19 isolates that were previously sequenced using an Illumina-only workflow and 65 isolates collected from intensive care unit patients across six Belgian hospitals. Besides direct comparison of the performance of Nanopore versus Illumina WGS data, this allowed us to investigate intra-patient heterogeneity, genotype-phenotype AMR concordance, plasmid identification, and the detection of genes encoding virulence factors.

## MATERIALS AND METHODS

### Collection of bacterial isolates

*P. aeruginosa* isolates from respiratory samples were collected between 10 July 2024 and 8 April 2025 from patients in ICUs in the following hospitals: Jessa Ziekenhuis (Hasselt), Ziekenhuis aan de Stroom (ZAS, Antwerpen), Imelda Ziekenhuis (Bonheiden), Jan Yperman Ziekenhuis (JYZ, Ieper), AZ Sint-Lucas (AZSL, Gent), and Onze-Lieve-Vrouwziekenhuis (AZORG, Aalst). Isolate AZORG_P5_PA1 was not included because the genome coverage was <40×.

### Bacterial cultures

Approximately half of a 10 µL disposable inoculation loop (Copan) was filled with colonies from a fresh overnight culture grown on Columbia agar with 5% sheep blood (BD) and subsequently transferred to 450 µL of DNA/RNA Shield (Zymo Research) and briefly vortexed to obtain a homogenous suspension.

### Antimicrobial susceptibility testing

Phenotypic susceptibility testing was performed per center as follows: AZORG: BD Phoenix (BD Diagnostic Systems) using the NMIC-408 panel; AZSL, Jessa: BD Phoenix (BD Diagnostic Systems) using the NMIC-474 panel; and Imelda, JYZ, ZAS: VITEK 2 (bioMérieux, Marcy-l'Etoile, France) using the AST-N446 card.

Interpretation of susceptibility testing results was based on the Clinical and Laboratory Standards Institute (CLSI) guidelines, 34^th^ ed. (2024) (13). The intermediate “I” category was omitted from the AMR genotype versus phenotype concordance calculations.

### DNA isolation and Nanopore whole-genome sequencing

Genomic DNA was isolated from the previously mentioned suspensions of plate-grown bacterial cultures in DNA/RNA shield using the RSC Cultured Cells DNA kit on the Maxwell RSC instrument (Promega) according to the manufacturer’s instructions. DNA concentration was assessed using the Qubit fluorometer dsDNA HS Assay kit (2765741, Thermo Fisher Scientific). The Rapid Barcoding kit 96 V14 (SQK-RBK114.96) from Oxford Nanopore Technologies (ONT) was used for the preparation of WGS libraries using 200 ng of extracted DNA per barcode as input material. Sequencing was performed on MinIon R10.4.1 flow cells, and reads were basecalled in real-time in super-accuracy mode using MinKNOW version 24.06.16. Libraries consisted of 8–14 samples per run and were sequenced for 63 h on average (range: 27–72 h), resulting in an average yield of 9.3 ± 3.5 Gigabases per flow cell or 900 ± 300 Megabases per sample (Table S1). A minimum sequencing coverage of 40× was required. In addition, the genomes of 19 *P. aeruginosa* ICU isolates that were previously sequenced via Illumina by De Geyter et al. and Moretti et al. (14, 15) were sequenced and analyzed using the Nanopore-only workflow described above.

### Bioinformatic analysis

BugSeq (version 2025-06-02) was used for Multilocus Sequence Typing (MLST) profiling, plasmid detection, AMR marker identification, and subsequent prediction of resistance based on CLSI criteria. In addition, this software was utilized for outbreak analysis by submitting basecalled ONT reads to the platform. The following genes were used to determine the MLST profile of *P. aeruginosa* isolates: *acsA* (locus 1), *aroE* (locus 2), *guaA* (locus 3), *mutL* (locus 4), *nuoD* (locus 5), *ppsA* (locus 6), and *trpE* (locus 7). Based on the specific combination of alleles, isolates are assigned to a particular sequence type (ST).

Furthermore, the Long Read Module of MBioSEQ Ridom Typer Client version 11.0.2 (2025-7) (Bruker) was used for genome assembly, core genome MLST (cgMLST), single-nucleotide variant (SNV) analysis, plasmid detection, and AMR identification using raw read data (16). Minimum spanning trees (MSTs; based on cgMLST data) and epicurves were also generated using this software. The MST cluster distance threshold for *P. aeruginosa* is 12 cgMLST alleles (17). Isolates that have an equal or lesser allelic difference than this threshold are part of the same complex type.

Virulence factors were predicted via VFDB 2.0 (18), while *in silico* serotyping was performed via PAST 2.0 (19) and has previously shown to successfully assign serogroup assignments in 99.27% of cases (20).

To investigate whether the large allelic differences between Illumina and ONT data for PAUZB069 were caused by methylation, the pod5 files for this isolate were re-basecalled using the v6.0.0 hac model (Oxford Nanopore Technologies), including the methylation model Modkit described in the pipeline of Galeone et al. (21). Next, the mean of 6mA-methylated positions was determined per locus for loci that were different between ONT and Illumina, as well as for loci that were identical between both platforms.

### Statistical analysis

A chi-square analysis using GraphPad (version 10.2.0) was performed to investigate the relationship between ST and serotype. The difference between the mean of 6mA-methylated positions per locus for loci that were different between ONT and Illumina versus those that were identical between both platforms was determined using Cohen’s *d* effect size measure. Since differing loci were also found to be longer, this confounder was corrected by calculating the density per kilobase (kB) and running a Wilcoxon signed-rank test for length-matched controls. The 95% confidence intervals for categorical agreement between AMR genotype and phenotype were calculated using the Clopper-Pearson exact method.

## RESULTS

### Pairwise comparison of Nanopore-only versus Illumina-only WGS data

**Fig 1:**
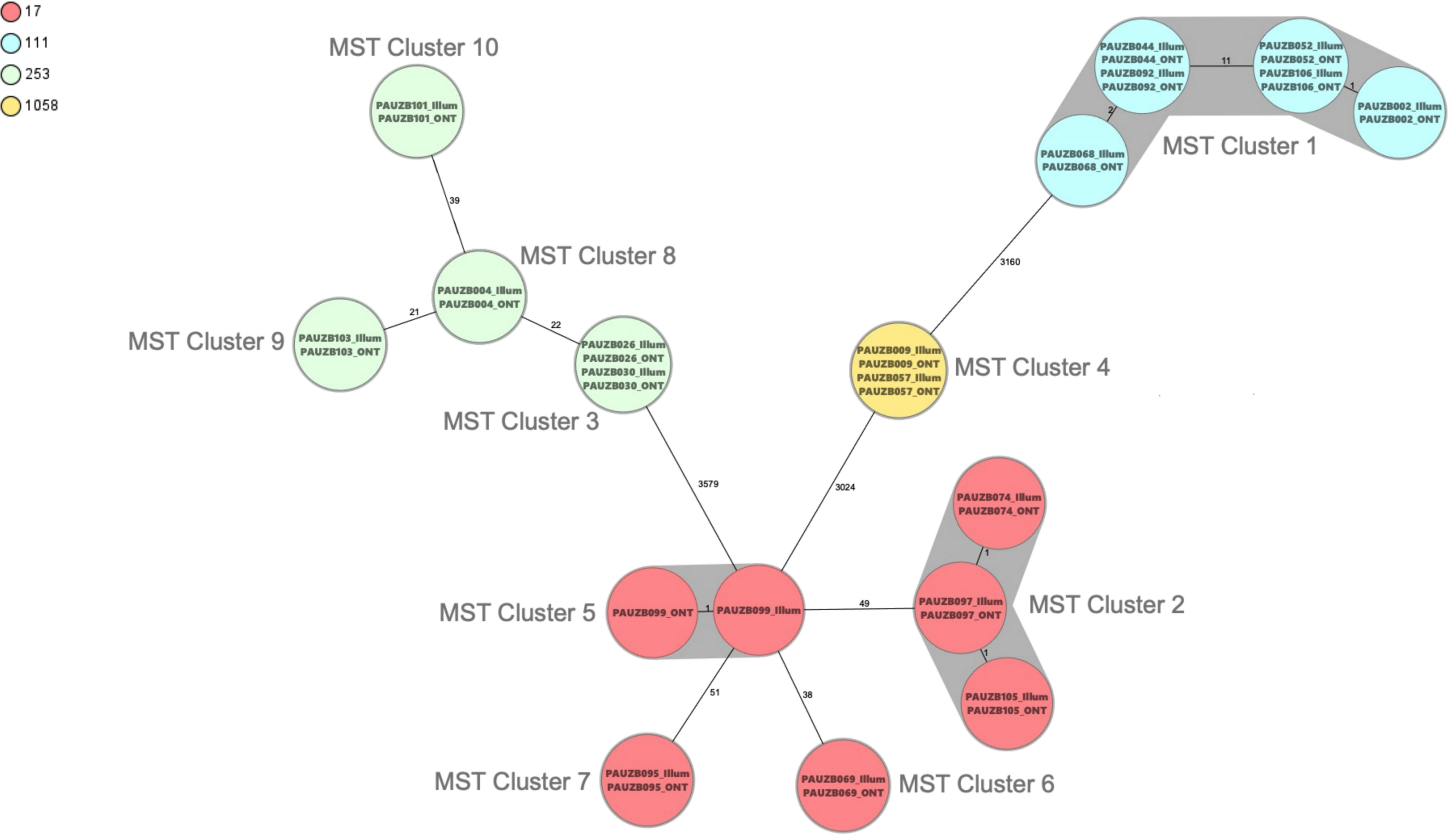
MST generated using the MBioSEQ Ridom Typer (Bruker) software based on cgMLST analysis of Illumina and Nanopore WGS data of 19 *P. aeruginosa* ICU strains. The distance was based on 3,867 cgMLST alleles (missing values were ignored pairwise) and is indicated on the lines interconnecting the nodes, which are colored according to sequence type. The MST cluster distance threshold was 12 cgMLST alleles.

A subset of 19 *P. aeruginosa* isolates from previously published studies that used Illumina sequencing to characterize strains associated with nosocomial transmission in intensive care units within a single Belgian hospital (14, 15) was sequenced using the Nanopore-only workflow (Table 1). When analyzing both the Illumina and Nanopore data sets using the BugSeq bioinformatic platform that utilizes refMLST (22) for outbreak analysis, there was high concordance both between both platforms, with 18 out of 19 Illumina/Nanopore pairs clustered together within a maximum distance of 10 refMLST alleles (Fig. S1). However, for one isolate (PAUZB069) belonging to sequence type (ST)17, a difference of 108 refMLST alleles was observed. Methylation analysis only identified 6mA methylation (no 4mC or 5mC methylation) and revealed that the mean number of 6mA-methylated positions per locus was about twice as high for loci that were different between Nanopore and Illumina compared to those that were identical for the PAUZB069 isolate (Table 2). When analyzing the same data sets with MBioSEQ Ridom Typer (Bruker), all 19 Illumina/Nanopore pairs clustered within a maximum difference of one cgMLST allele (Fig. 1). In addition, all outbreak clusters previously identified with Illumina sequencing were confirmed using the Nanopore-only workflow (Table 1). There was 100% concordance between both platforms at the level of AMR prediction, identification of virulence factor genes, and serotype (Table 3; Table S5). However, for plasmid identification, considerable differences were observed depending on the bioinformatics platform used, as BugSeq showed complete concordance in plasmid identification for 17/19 isolates, while MBioSEQ Ridom Typer only showed complete concordance for 6/19 isolates (Table 3). Nevertheless, all identified conjugative plasmids (circular using Nanopore assembly) were correctly identified by both platforms regardless of the bioinformatic pipeline, whereas many of the non-circular plasmids (AE985, AF876, AB961, and AE974) predicted using the MBioSEQ Ridom Typer software seemed to map to regions of genomic plasticity on the chromosome, as for instance, the genes encoding the type II secretion system were part of the AE985 plasmid only predicted using Illumina in combination with MBioSEQ Ridom Typer (Table S5).

**TABLE 1:** Overview of the cluster identification based on Illumina- and Nanopore-only sequencing of 19 *P. aeruginosa* ICU isolates, selected from a previously published data set (14, 15)^*b*^

Isolate	Collection date	ST	Serotype	Illumina cluster^*a*^	Nanopore cluster
PAUZB002	27/08/2019	111	O12	1	1
PAUZB052	30/11/2019	111	O12	1	1
PAUZB106	8/12/2020	111	O12	1	1
PAUZB044	22/06/2019	111	O12	1	1
PAUZB068	1/10/2019	111	O12	1	1
PAUZB092	9/04/2019	111	O12	1	1
PAUZB074	10/07/2019	17	O1	2	2
PAUZB097	14/02/2019	17	O1	2	2
PAUZB105	28/09/2020	17	O1	2	2
PAUZB099	14/05/2019	17	O1	Singleton	Singleton
PAUZB069	14/08/2019	17	O1	Singleton	Singleton
PAUZB095	17/05/2019	17	O1	Singleton	Singleton
PAUZB026	28/08/2019	253	O10	3	3
PAUZB030	7/10/2019	253	O10	3	3
PAUZB004	27/08/2019	253	O10	Singleton	Singleton
PAUZB101	1/07/2019	253	O10	Singleton	Singleton
PAUZB103	22/08/2019	253	O10	Singleton	Singleton
PAUZB009	27/08/2019	1058	O6	4	4
PAUZB057	23/08/2019	1058	O6	4	4

^*a*^ The cluster numbers do not correspond to those in the original publications from Degeyter et al. and Moretti et al.

^*b*^ Cluster identification was performed using the MBioSEQ Ridom Typer (Bruker) software that utilizes an MST cluster distance threshold of 12 cgMLST alleles.

**TABLE 2:** Difference in level of 6mA methylation between loci that are different between ONT and Illumina versus those that are identical for PAUZB069 using BugSeq analysis^*a*^

ONT vs Illumina loci	*n*	Mean 6mA/locus	% loci with ≥1 6mA	% loci with ≥2 6mA
Differing	93	2.23	94.6	51.6
Identical	5,097	0.92	49.3	23.7

^*a*^ Cohen’s *d* = 1.01 (large). Since differing loci are also longer (a confounder), the test was corrected via calculating the density per kb (*p* = 2.8 × 10^−16^, *d* = 0.73) as well as by running a length-matched control (±10% length; 2.23 versus 1.29, Wilcoxon *p* = 1.7 × 10^−8^).

**TABLE 3:** Identified plasmids, predicted AMR, and serotype concordance for Illumina versus Nanopore WGS

Isolate	Plasmids via BugSeq		Plasmids via MBioSeq RidomTyper		Predicted AMR concordant?	Predicted virulence factor genes concordant?
	Illumina	ONT	Illumina	ONT		
PAUZB002	AG036^*a*^	AG036^*a*^	AG036^*a*^, AE985	AG036^*a*,*b*^	Yes	Yes
PAUZB052	AG036^*a*^	AG036^*a*,*b*^	AG036^*a*^, AE985	AG036^*a*,*b*^	Yes	Yes
PAUZB106	AG036^*a*^	AG036^*a*^	AG036^*a*^, AE985, AF876	AG036^*a*^^,*b*^; AF876	Yes	Yes
PAUZB044	None	None	AE985, AB961	None	Yes	Yes
PAUZB068	None	None	AE985, AB961	None	Yes	Yes
PAUZB092	None	None	AE985	None	Yes	Yes
PAUZB074	AG036^*a*^; AG216	AG036^*a*^^,*b*^	AG036^*a*^, AG216	AG036^*a*,*b*^	Yes	Yes
PAUZB097	AG036^*a*^; AG216	AG036^*a*^^,*b*^, AG216	AG036^*a*^, AG216	AG036^*a*^^,*b*^, AG216, AF876	Yes	Yes
PAUZB105	AG036^*a*^; AG216	AG036^*a*,*b*^	AG036^*a*^, AG216, AE985	AG036^*a*^^,*b*^, AF876	Yes	Yes
PAUZB099	None	None	AF876	AF876	Yes	Yes
PAUZB069	None	None	AE985	AF876	Yes	Yes
PAUZB095	AC882^*b*^	AC882^*b*^	AC882, AE974	AC882	Yes	Yes
PAUZB026	None	None	None	None	Yes	Yes
PAUZB030	None	None	None	None	Yes	Yes
PAUZB004	None	None	None	None	Yes	Yes
PAUZB101	None	None	AE985	AC907	Yes	Yes
PAUZB103	None	None	AF876	None	Yes	Yes
PAUZB009	None	None	None	None	Yes	Yes
PAUZB057	None	None	None	None	Yes	Yes

^*a*^ Conjugative plasmid.

^*b*^ Circular plasmid.

### Clustering of longitudinally collected *P. aeruginosa* isolates

**Fig 2:**
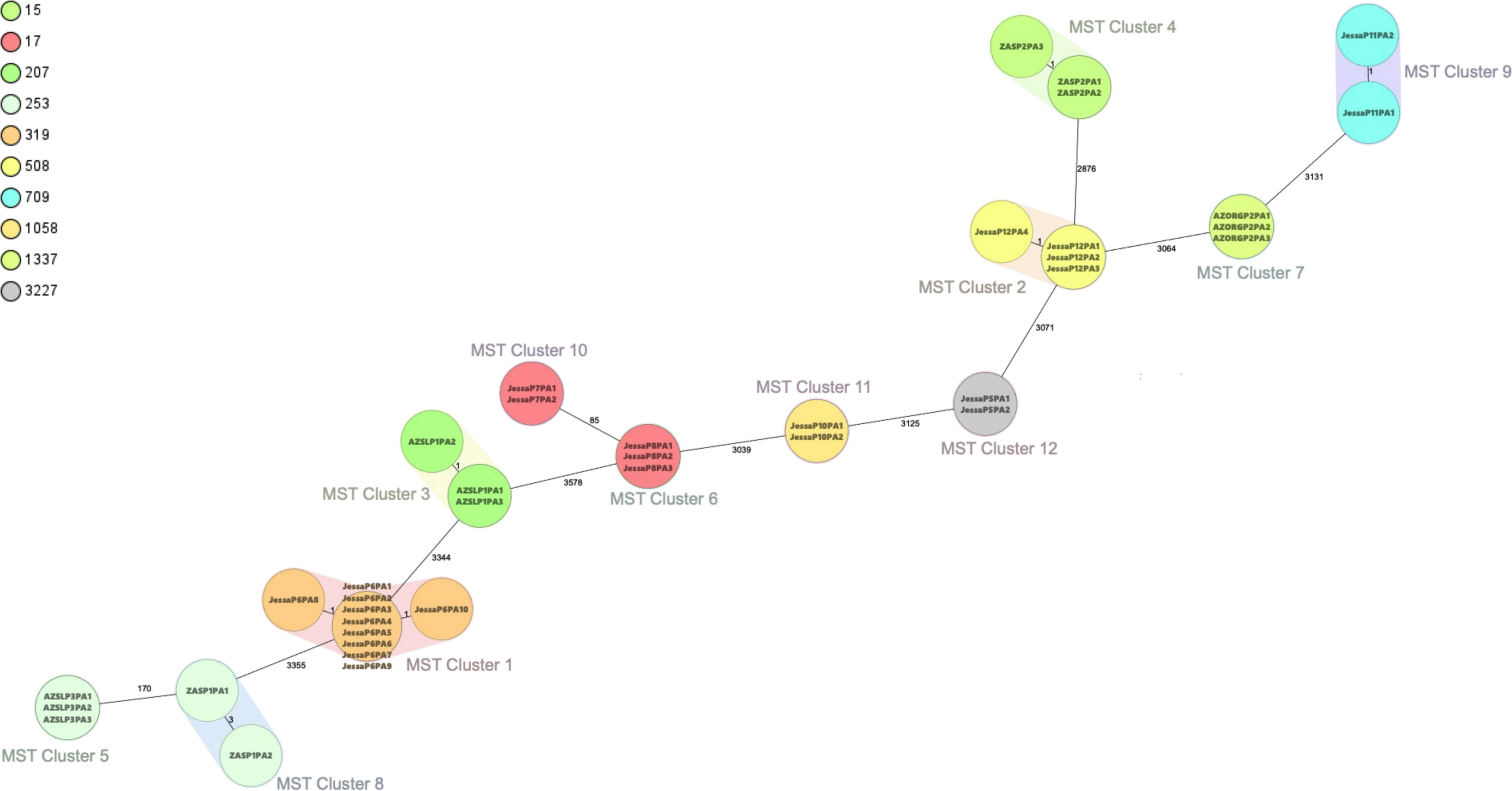
MST based on cgMLST analysis of 39 longitudinally collected *P. aeruginosa* isolates using the MBioSEQ Ridom Typer (Bruker) software. The distance was based on 3,867 cgMLST alleles (missing values were ignored pairwise) and is indicated on the lines interconnecting the nodes, which are colored according to sequence type. The MST cluster distance threshold was 12 cgMLST alleles.

To determine whether within-host heterogeneity and short-term evolution of *P. aeruginosa* could interfere with outbreak analysis interpretation, 39 isolates collected from 12 different patients and belonging to 10 different STs were sequenced using the Nanopore-only workflow and analyzed via MBioSEQ Ridom Typer (Bruker) software (Fig. 2; Table 4; Table S3). A maximum difference of three cgMLST alleles was observed between isolates collected from the same patient. When investigating the SNVs that resulted in the allelic differences, it was found that 11 out of 12 SNVs comprised non-synonymous mutations that occurred in a total of eight *P. aeruginosa* genes belonging to the core genome (*fliJ*, *ampD*, *rmlB*, *gcvT1*, *anr*, *recC*, *nfxB*, and *PA5082*; Table S3).

**TABLE 4:** Summary of the *P. aeruginosa* isolates collected in this multicenter study

Isolate	Collection date	Genome size (Mbp)	ST	Complex type	MST Cluster^*a*^	Serotype
AZORGP1PA1	30/01/2025	7.0	381	7618	Singleton	O2
AZORGP2PA1	17/02/2025	6.4	1337	7619	7	O6
AZORGP2PA2	24/02/2025	6.5	1337	7619	7	O6
AZORGP2PA3	27/02/2025	6.4	1337	7619	7	O6
AZORGP3PA1	22/01/2025	6.4	703	7621	Singleton	O6
AZORGP4PA1	23/01/2025	6.5	485	7622	Singleton	O4
AZORGP6PA1	7/04/2025	7.1	395	7623	Singleton	O6
AZORGP7PA1	7/03/2025	6.7	319	7624	Singleton	O11
AZORGP8PA1	8/04/2025	7.2	244	7625	Singleton	O5
AZSLP1PA1	23/12/2024	6.5	207	2617	3	O1
AZSLP1PA2	30/12/2024	6.5	207	2617	3	O1
AZSLP1PA3	6/01/2025	6.5	207	2617	3	O1
AZSLP2PA1	6/01/2025	6.7	253	494	Singleton	O10
AZSLP3PA1	12/02/2025	7.0	253	7626	5	O10
AZSLP3PA2	24/02/2025	7.0	253	7626	5	O10
AZSLP3PA3	5/03/2025	7.0	253	7626	5	O10
AZSLP4PA1	7/02/2025	7.0	319	7627	Singleton	O11
AZSLP5PA1	30/03/2025	6.5	1750	7628	Singleton	O3
ImeldaP1PA1	14/01/2025	6.9	245	7629	Singleton	O2
ImeldaP2PA1	21/01/2025	6.4	3996	7630	Singleton	O6
ImeldaP3PA1	23/01/2025	6.5	500	7631	Singleton	O6
ImeldaP4PA1	2/04/2025	7.4	313	492	Singleton	O1
JessaP1PA1	10/07/2024	6.5	646	7635	Singleton	O7
JessaP2PA1	17/07/2024	6.7	646	7636	Singleton	O7
JessaP3PA1	22/07/2024	6.6	2069	2741	Singleton	O6
JessaP4PA1	16/10/2024	6.4	313	3203	Singleton	O1
JessaP5PA1	17/10/2024	6.4	3227	7637	12	O3
JessaP5PA2	24/10/2024	6.4	3227	7637	12	O3
JessaP6PA1	24/11/2024	6.7	319	7638	1	O11
JessaP6PA2	2/12/2024	6.7	319	7638	1	O11
JessaP6PA3	2/12/2024	6.8	319	7638	1	O11
JessaP6PA4	10/12/2024	6.6	319	7638	1	O11
JessaP6PA5	12/12/2024	6.7	319	7638	1	O11
JessaP6PA6	19/12/2024	6.6	319	7638	1	O11
JessaP6PA7	23/12/2024	6.7	319	7638	1	O11
JessaP6PA8	26/12/2024	6.6	319	7638	1	O11
JessaP6PA9	2/01/2025	6.7	319	7638	1	O11
JessaP6PA10	9/01/2025	6.7	319	7638	1	O11
JessaP7PA1	12/12/2024	6.7	17	7639	10	O1
JessaP7PA2	26/12/2024	6.7	17	7639	10	O1
JessaP8PA1	4/01/2025	7.1	17	7640	6	O1
JessaP8PA2	4/01/2025	7.2	17	7640	6	O1
JessaP8PA3	6/01/2025	7.1	17	7640	6	O1
JessaP9PA1	10/02/2025	6.7	253	4268	Singleton	O10
JessaP10PA1	12/02/2025	6.5	1058	7632	11	O6
JessaP10PA2	13/02/2025	6.5	1058	7632	11	O6
JessaP11PA1	13/02/2025	7.3	709	7633	9	O5
JessaP11PA2	20/02/2025	7.3	709	7633	9	O5
JessaP12PA1	24/03/2025	6.4	508	7634	2	O3
JessaP12PA2	26/03/2025	6.4	508	7634	2	O3
JessaP12PA3	26/03/2025	6.4	508	7634	2	O3
JessaP12PA4	27/03/2025	6.4	508	7634	2	O3
JYZP1PA1	24/01/2025	6.8	463	7642	Singleton	O4
JYZP2PA1	21/03/2025	6.8	244	424	Singleton	O5
JYZP3PA1	30/03/2025	6.8	532	7643	Singleton	O11
ZASP1PA1	13/12/2024	6.7	253	7648	8	O10
ZASP1PA2	17/01/2025	6.8	253	7648	8	O10
ZASP2PA1	17/12/2024	6.8	15	7649	4	O3
ZASP2PA2	17/12/2024	6.8	15	7649	4	O3
ZASP2PA3	27/12/2024	6.8	15	7649	4	O3
ZASP3PA1	25/12/2024	7.4	179	7650	Singleton	O6
ZASP4PA1	11/01/2025	6.8	381	2729	Singleton	O5
ZASP5PA1	12/01/2025	6.3	/^*b*^	7653	Singleton	O3
ZASP6PA1	9/02/2025	6.4	262	7651	Singleton	O6
ZASP7PA1	22/02/2025	6.7	274	7655	Singleton	O3

^*a*^ In analysis of longitudinally collected isolates.

^*b*^ Unknown sequence type.

### Multicentric *P. aeruginosa* outbreak investigation

**Fig 3:**
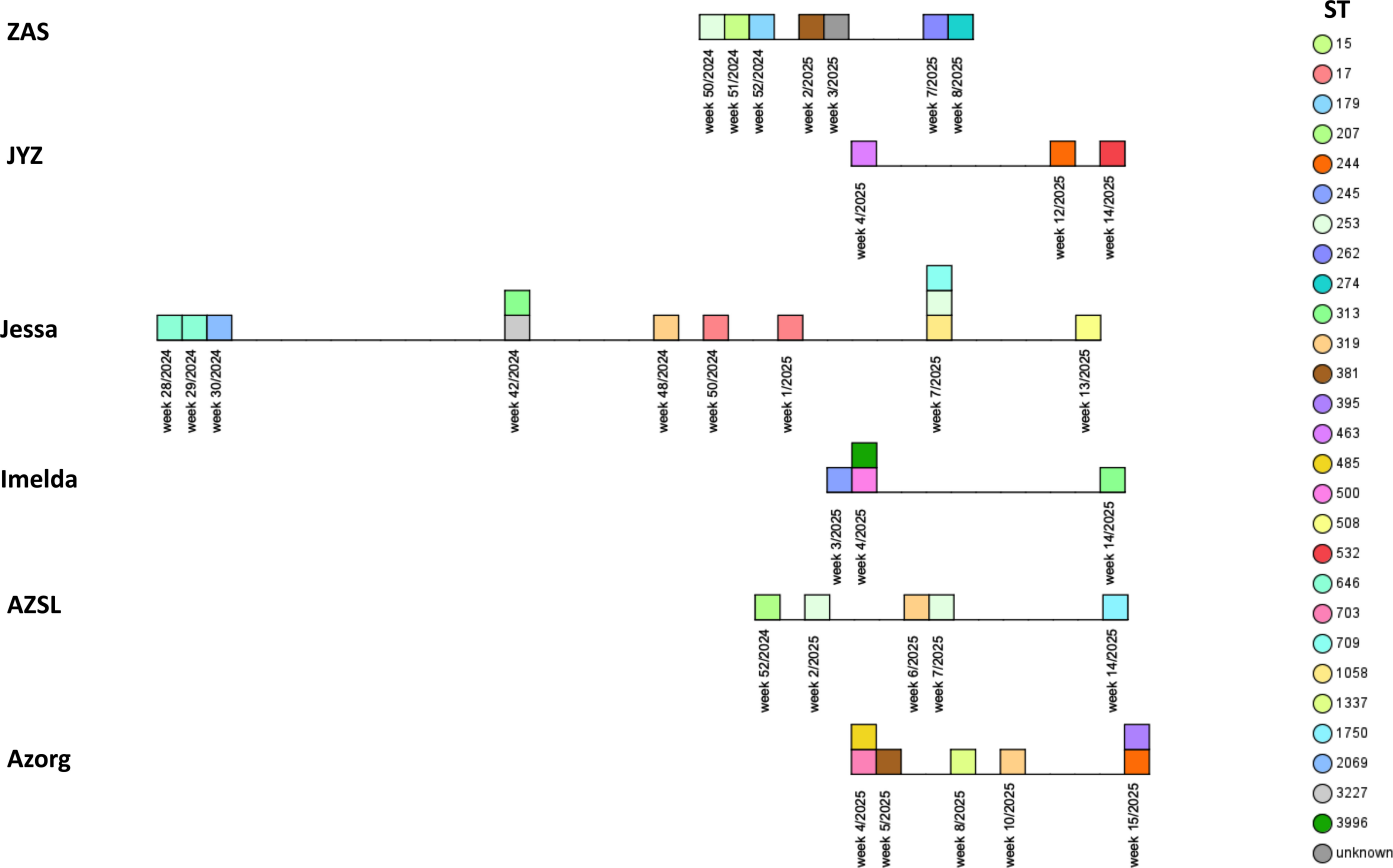
Epidemiological curves of the different *P. aeruginosa* isolates that were collected at six Belgian ICUs during the multicentric study period. Only the first isolate per patient was visualized. *P. aeruginosa* isolates are represented by cubes, which are colored according to sequence type (see legend on the right).

**Fig 4:**
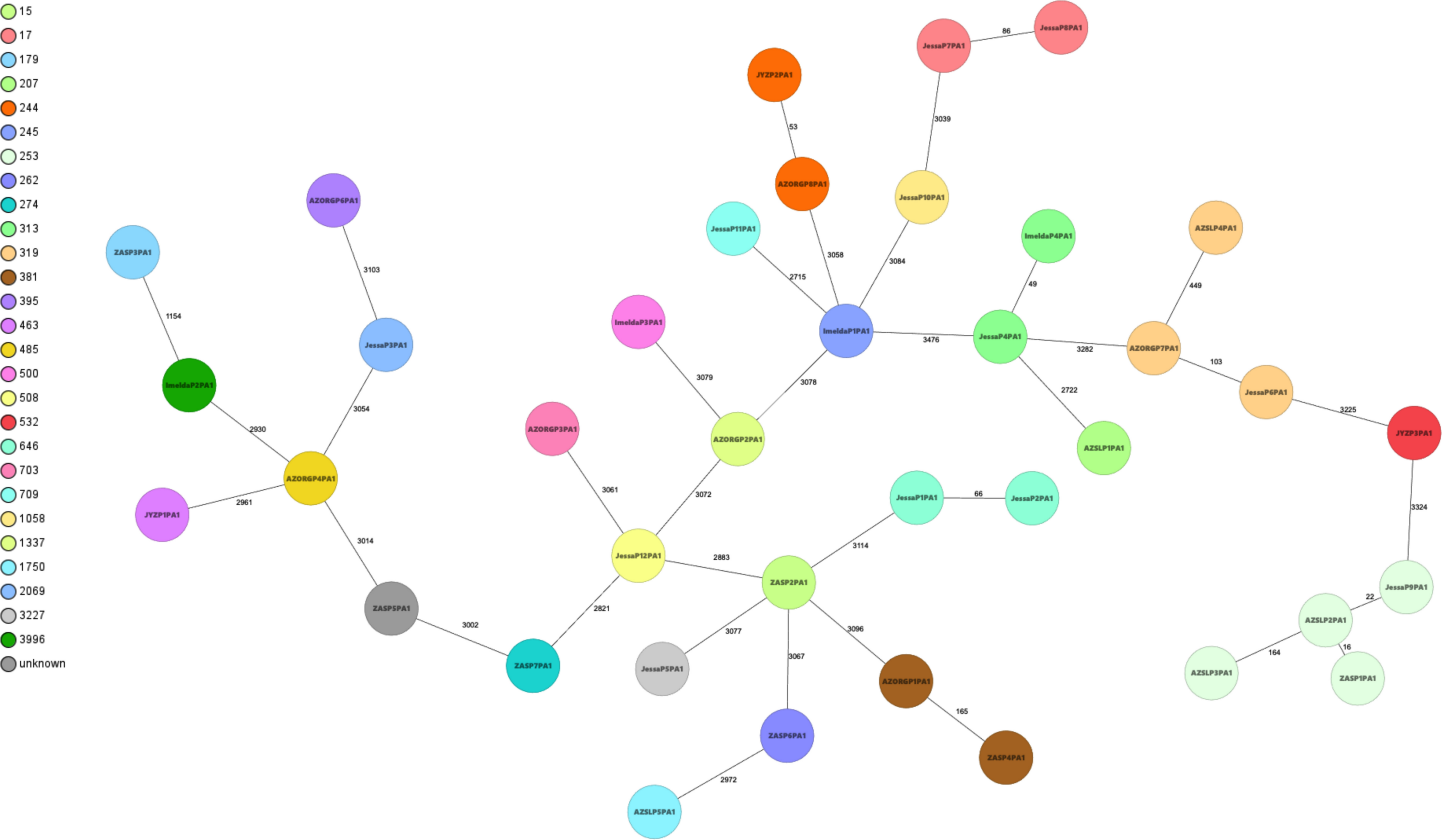
MST based on cgMLST analysis of 38 *P. aeruginosa* isolates (one per patient) collected in the multicentric study comprising six Belgian ICUs. The distance was based on 3,867 cgMLST alleles (missing values were ignored pairwise) and is indicated on the lines interconnecting the nodes, which are colored according to sequence type. The MST cluster distance threshold was 12 cgMLST alleles. The analysis was performed using the MBioSEQ Ridom Typer (Bruker) software.

Since all longitudinally collected isolates clustered together, one isolate per patient was selected for outbreak investigation across six Belgian ICUs, totaling 38 isolates (Fig. 3). Although certain STs were found in multiple hospitals (ST244, ST17, ST253, ST313, ST319, and ST381) and even within the same ICU (ST17, ST253, and ST646) during the study period, no transmission clusters were identified (Fig. 3 and 4; Table 4). SNV analysis showed that isolates that belonged to the same sequence type but did not cluster together could be distinguished from each other by several mutations (no indels) in genes encoding virulence factors that are distributed across the *P. aeruginosa* genome (Table S4).

### Virulence factors, serotypes, and plasmids

All isolates harbored genes for the biosynthesis of type IV pili, alginate, lipopolysaccharide (LPS), and alkaline protease (Table S5). In 12 out of 65 isolates (18.46%) corresponding to 3 out of 38 patients (7.9%), the exotoxin A encoding gene was not detected. Both genes that are part of the type II secretion system (T2SS) and type III secretion system (T3SS) were detected, along with their respective effectors (Table S5). Although the T3SS effector toxin *exoT* was detected in all isolates, *exoY* was detected in 96.9% of isolates (94.7% of patients), while *exoS* (61.5% of isolates; 71.1% of patients) and *exoU* (36.9% of isolates; 26.3% of patients) showed a mutually exclusive presence as expected. Iron acquisition system genes, including pyoverdine, pyochelin, and pyocyanin, were uniformly present. Additional virulence-associated system genes, such as those involved in the Xcp secretion system and flagellar biosynthesis, were also observed in all isolates. Furthermore, all isolates harbored genes encoding the HSI-1 type VI secretion system (T6SS) and its associated effectors (*tse1*, *tse2*, and *tse3*). Genes related to quorum sensing, rhamnolipid biosynthesis, and phospholipase C production were also observed in all isolates.

Serotype O6 (23.7%) was most represented among patients, followed by serotypes O3 (15.8%), O1 (13.2%), O5 (10.5%), O10 (10.5%), O11 (10.5%), O2 (5.3%), and O7 (5.3%). There was a significant association between ST and serotype (*P* < 0.05), as only ST381 was associated with two different serotypes (O2 and O5) (Table S5).

Plasmid analysis using BugSeq revealed the absence of plasmids in 62 out of 65 isolates (95.4%) (Table 5). AC882 was detected in two isolates (Imelda_P4_PA1 and ZAS_P3_PA1), and AC068 was detected in one isolate (Jessa_P6_PA9). None of these plasmids carried AMR markers. When using MBioSEQ Ridom Typer (Bruker), 13 additional plasmids were identified as 10 out of 65 (15.4%) of isolates harbored at least one plasmid (Table 5). The four plasmids belonging to primary cluster ID AA379 harbored fluoroquinolone resistance genes. None of the plasmids were mobilizable (Table S6).

**TABLE 5:** Overview of the plasmids detected among the 65 *P. aeruginosa* isolates from the multicentric study using MBioSEQ Ridom Typer (Bruker)^*a*^

Plasmid	Size (bp)	Primary cluster ID	Secondary cluster ID	AMR	Rep type(s)	BugSeq
p1_AZORGP2PA2	62,251	AA379	AI397	None		No
p2_AZORGP2PA3	2,974	AA379	AI397	None		No
p1_JessaP8PA3	49,736	AA379	AI397	None		No
p1_AZORGP2PA3	7,552	AB990	AK993	None		No
p80.1_JessaP6PA9	80,116	AC068		None	IncP	Yes
p2_ImeldaP4PA1	212,684	AC882	AM239	None		Yes
p1_ZASP3PA1	212,833	AC882	AM239	None		Yes
p1_ImeldaP4PA1	242,629	AC907	AM268	None		No
p77.2_AZORGP1PA1	77,151	AF876		FQ: crpP/crpP/crpP	rep_cluster_322	No
p88.0_AZSLP3PA1	88,042	AF876		FQ: crpP	rep_cluster_322	No
p77.4_AZSLP3PA2	77,388	AF876		FQ: crpP	rep_cluster_322	No
p85.2_AZSLP3PA3	85,228	AF876		FQ: crpP	rep_cluster_322	No
p3_ImeldaP4PA1	85,442	AF917	AP925	None		No
p1_AZSLP3PA1	7,394	AH279	AR335	None		No
p1_AZSLP3PA2	7,418	AH279	AR335	None		No
p1_AZSLP3PA3	7,403	AH279	AR335	None		No

^*a*^ AMR, antimicrobial resistance; FQ, fluoroquinolone.

### Prediction of antimicrobial resistance

The overall categorical agreement (% [95% CI]) between AMR genotype and phenotype was 94.76% [92.46%–96.52%] (Table 6; Tables S7 to S9). Although high concordance (>95%) between predicted AMR genotype and phenotype was achieved for amikacin, tobramycin, cefepime, ceftazidime-avibactam, and levofloxacin, this was substantially lower for ciprofloxacin, piperacillin-tazobactam, ceftazidime, meropenem, and imipenem, as >3% very major errors were observed for those antibiotics.

**TABLE 6:** Categorical agreement between predicted AMR based on genotyping results and AMR phenotype^*a*^

Antibiotic	Categorical agreement (95% CI)	VME (95% CI)	ME (95% CI)
Amikacin	100.00% (94.31%–100.00%)	0.00% (0.00%–5.69%)	0.00% (0.00%–5.69%)
Tobramycin	100.00% (92.29%–100.00%)	0.00% (0.00%–7.71%)	0.00% (0.00%–7.71%)
Cefepime	100.00% (93.62%–100.00%)	0.00% (0.00%–6.38%)	0.00% (0.00%–6.38%)
Ceftazidime	91.23% (80.70%–97.09%)	8.77% (2.91%–19.30%)	0.00% (0.00%–6.27%)
Ceftazidime-avibactam	100.00% (88.06%–100.00%)	0.00% (0.00%–11.94%)	0.00% (0.00%–11.94%)
Imipenem	84.44% (70.54%–93.51%)	15.56% (6.49%–29.46%)	0.00% (0.00%–7.87%)
Meropenem	90.91% (80.05%–96.98%)	9.09% (3.02%–19.95%)	0.00% (0.00%–6.49%)
Piperacillin-tazobactam	91.23% (80.70%–97.09%)	7.02% (1.95%–17.00%)	1,.75% (0.04%–9.39%)
Ciprofloxacin	93.44% (84.05%–98.18%)	4.92% (1.03%–13.71%)	1.64% (0.04%–8.80%)
Levofloxacin	97.83% (88.47%–99.94%)	2.17% (0.06%–11.53%)	0.00% (0.00%–7.71%)
Overall	94.76% (92.46%–96.52%)	4.85% (3.17%–7.08%)	0.39% (0.05%–1.40%)

^*a*^ VME, very major error; ME, major error. AMR prediction according to BugSeq version 2025-06-02.

## DISCUSSION

Accurate WGS data are essential for reliable outbreak investigation. Illumina sequencing has been considered as the gold standard with regard to raw read accuracy exceeding Q30 (>99.9%) levels. However, recent advancements in ONT—especially with the R10.4 flow cell and V14 chemistry—have improved accuracy to about 99%, narrowing the gap with Illumina. With these improvements, ONT offers a competitive alternative, combining improved accuracy with the advantage of long-read data and near-real-time data acquisition (5–7). Indeed, in this study, we were able to provide WGS results in approximately 65 h from sample to result when sequencing an average of 11 *P. aeruginosa* genomes per run (Table S1), which could be reduced to approximately 24 h when running fewer than eight samples. When Nanopore-only WGS data of 19 previously sequenced *P. aeruginosa* isolates were compared to Illumina data and analyzed via BugSeq, there was high concordance between both platforms for most sequence types. However, for one isolate belonging to ST17, there was a difference of >100 refMLST alleles when comparing Nanopore versus Illumina data. Interestingly, the amount of 6mA-methylated positions was significantly higher at the differing alleles compared to identical alleles between ONT and Illumina for this particular isolate. A similar observation has been done by Dabernig-Heinz et al., in which strain-specific typing errors were identified in *Enterococcus faecium*, *Klebsiella pneumoniae*, *Listeria monocytogenes*, and *Staphylococcus aureus*, due to methylation-related errors at specific sites in the genome (7). Such incorrect reads occur at similar frequencies as correct reads in that position, resulting in random typing errors when the incorrect read reaches the majority threshold. When the same raw read data were analyzed with the Long Read Module of MBioSEQ Ridom Typer (Bruker), there was high concordance (maximum one cgMLST allele difference) between Nanopore and Illumina data for all strains and sequence types. A plausible explanation for this discrepancy is that the latter software uses an ONT cgMLST polisher during the data assembly step. In this process, basecalled FASTQ reads are mapped to the assembly consensus FASTA sequence using minimap2, followed by screening for positions in the core and accessory genome MLST genes that might be indicative of methylation-related sequencing errors, and ambiguous positions are masked with an “N” so that they are omitted during the cgMLST analysis. This ONT cgMLST polisher was previously evaluated using 80 multidrug-resistant organisms, including several *P. aeruginosa* isolates, and significantly lowered the allelic distance when comparing Nanopore-only data to that of an Illumina + Nanopore hybrid assembly (23). In another study, it was shown that Nanopore-only WGS is comparable with the performance obtained by Illumina data for outbreak investigation of *Corynebacterium diphtheriae*, but that issues with calling of methylated bases occurred for accurate outbreak investigation of vancomycin-resistant enterococci, which could be resolved using updated basecallers in combination with a PCR-based library preparation kit (24). This highlights the importance of the bioinformatic software to assemble and analyze bacterial genomes for accurate outbreak investigation. Besides bioinformatic software, the basecalling software can also have a significant effect on Nanopore-only WGS data, with the latest released basecalling models being trained to reduce errors resulting from bacterial methylation. Therefore, a combination of the latest basecalling model and bioinformatic software that can handle methylation-dependent sequencing errors is recommended for accurate outbreak investigation.

In the present study, we have not observed large genotypic heterogeneity, as longitudinally collected isolates from the same patient showed minimal allelic distances (maximum difference of three cgMLST alleles). Interestingly, SNVs comprised mostly non-synonymous mutations that occurred in genes related to motility (*fliJ* involved in flagellar biosynthesis), anaerobic adaptation (*anr*; anaerobic response regulator), multidrug efflux (*nfxB*; repressor of mexCD-oprJ efflux pump), and antibiotic resistance (*ampD*; negative regulator of AmpC β-lactamase expression). It is likely that these mutations occur as a consequence of host adaptation rather than being random sequencing errors introduced via the Nanopore methodology. Previous studies have shown that *P. aeruginosa* can exhibit significant genotypic and phenotypic heterogeneity in patients with cystic fibrosis, COPD, or bronchiectasis, in particular when colonizing patients for longer periods of time (25–29). The limited amount of genetic heterogeneity in our study might be explained by the relatively short time period in which longitudinal isolates were collected, as well as by the patient population, which is different from the aforementioned studies. Nevertheless, the minimal allelic differences (maximum three cgMLST alleles) between longitudinal *P. aeruginosa* isolates from the same patient are well below the cut-off value for genetic clustering (12 cgMLST alleles), indicating that the observed heterogeneity does not interfere with accurate outbreak analysis.

*P. aeruginosa* infections are difficult to treat owing to their high intrinsic antibiotic tolerance or resistance via the presence of genes encoding efflux pumps, the production of virulence factors, and its rapid adaptation to infection (30, 31). In this study, the categorical agreement between genotypic predictions and phenotypic antibiotic susceptibility profiles was >80% for each tested antibiotic and ca. 95% overall. This is in line with the studies of Ferreira et al. and Cunningham et al., which reported overall categorical agreements of 85.4% and 92.4%, respectively (32, 33), but remarkably higher than the 52.4% overall categorical agreement reported by Harris et al., although the latter study performed shotgun metagenomics directly on positive blood cultures (34). When looking at specific antibiotics, similarly high levels of categorical agreement were found for amikacin (97.8%–100%) and tobramycin (92.6%–100%), whereas ceftazidime (0%–86.8%) and piperacillin-tazobactam (76.3%–84.2%) consistently showed the lowest levels of categorical agreement when comparing this study to the aforementioned studies (32–34). Indeed, high percentages (>3%) of very major errors were observed among cephalosporins and carbapenems in this study. Analysis of mutations in longitudinally collected isolates that showed a very major error revealed that isolate JessaP6PA10 had a mutation in the *nfxB* gene (Table S3), which is known to affect ciprofloxacin resistance through upregulation of the MexCD-OprJ efflux pump (35), whereas isolate ZASP2PA3 had a mutation in the *ampD* gene (Table S3), which acts as a negative regulator of the chromosomal AmpC β-lactamase and is associated with piperacillin-tazobactam resistance (36). Nevertheless, other very major errors seemed to be strain-related and could not be explained to the point mutation level, indicating that differences in intrinsic resistance between genetically similar *P. aeruginosa* strains or isolates might affect the performance of AMR prediction algorithms, possibly due to the complex regulatory mechanisms of membrane permeability and multidrug efflux in *P. aeruginosa* as previously reported (37). Indeed, it has been shown that *P. aeruginosa* can exhibit significant phenotypic heterogeneity despite genetic similarity. Studies on cystic fibrosis isolates, for example, have shown that closely related genotypes can display diverse resistance profiles (38, 39)

Analysis of *P. aeruginosa* isolates in this multicenter study revealed the presence of a broad array of genes encoding virulence factors, underscoring the organism’s multifaceted pathogenic potential. Consistent with previous studies, all isolates harbored genes related to type IV pili, alginate, and LPS, critical for adhesion, biofilm formation, and immune evasion. The near-universal presence of genes encoding exotoxin A and the T3SS with different combinations of its effectors (*exoS*, *exoT*, *exoY*, and *exoU*) suggests heterogeneity in cytotoxic and immunomodulatory capabilities across our isolates (40). While *exoT* and *exoY* are detected in most strains, *exoS* and *exoU* are nearly mutually exclusive (41, 42). ExoU possesses potent phospholipase activity, which causes rapid cell lysis and necroptosis of mammalian cells. Furthermore, ExoU, considered the most toxic effector protein delivered by the T3SS, is associated with greater virulence and more severe clinical outcomes in human infections (43–45). ST253, also observed in this study, includes the virulent strain PA14 possessing *exoU* (46). Furthermore, ST17 and ST253 are described as widespread clones (30). High-risk clones, such as ST235, ST111, and ST175 that are globally disseminated and are frequently linked to outbreaks with MDR strains, were not observed in this study (30). In a study of *Escherichia coli* populations in the UK, higher antimicrobial usage was associated with an increased prevalence and success of MDR strains (47). Together, these findings confirm the adaptive versatility and high virulence potential of *P. aeruginosa*, aligning with its known clinical significance as a versatile and opportunistic pathogen (40, 46). Serotype analysis revealed a statistically significant association between ST and serotype. Although certain STs were linked to specific serotypes, individual STs could also contain multiple serotypes, indicating genomic plasticity and possible antigenic variation or recombination at O-antigen loci. O6 and O11, also present in the isolates, are the most prevalent serotypes among invasive *P. aeruginosa* isolates and are also prevalent in critically ill patients (48). Furthermore, some serotypes are more virulent than others; serotype O11 has been linked to enhanced virulence, particularly due to its more frequent expression of *exoU* (49). Plasmids, which are important vehicles for the transfer of AMR and virulence genes, were only detected in a minority of isolates in this study, and only one of them carried an AMR marker. In a comparable study, plasmids were also found to be relatively rare (29, 50), but more common among MDR high-risk clones.

One limitation of this study is that the prospective study was not carried out in a true outbreak scenario, although the ability to identify transmission clusters was validated using retrospective isolates from a previous outbreak. Another limitation can be found in the fact that only a limited number of patients and isolates were included in a relatively short time period. Finally, due to its multicentric nature, analytical variability in antimicrobial susceptibility testing might have affected the observed genotype-phenotype concordance.

### Conclusion

Nanopore-only WGS using the Rapid Barcoding kit in combination with bioinformatic software that can reduce methylation errors in cgMLST analysis enables high-resolution outbreak investigation and AMR prediction for *P. aeruginosa* in the ICU setting, with results consistent with established Illumina-based methods.

## Data Availability

Assembled *P. aeruginosa* genomes using MBioSEQ Ridom Typer (Bruker) were submitted to NCBI under the BioProject accession number PRJNA1438213. All data for the cgMLST analysis are included in the supplemental materials. The pipeline script for genome assembly and raw sequencing data are available upon request.

## SUPPLEMENTAL MATERIAL

Supplemental legends jcm.00572-26-s0001.docx Legends for Tables S1 to S9 and Fig. S1.

Table S1 jcm.00572-26-s0002.xlsx Overview of the number of samples, the duration of sequencing and the total yield (successfully called Gigabases) per Nanopore WGS run.

Table S2 jcm.00572-26-s0003.xlsx Overview of the core genome allelic profile of all *P. aeruginosa* isolates in the multicentric study.

Table S3 jcm.00572-26-s0004.xlsx Overview of SNVs in longitudinally collected *P. aeruginosa* isolates.

Table S4 jcm.00572-26-s0005.xlsx Pairwise comparison of SNVs between isolates from different patients belonging to the same sequence type.

Table S5 jcm.00572-26-s0006.xlsx Overview of genes encoding virulence factors and the predicted serotype amongst *P. aeruginosa* isolates collected in this study.

Table S6 jcm.00572-26-s0007.xlsx Detailed overview of plasmids in this study.

Table S7 jcm.00572-26-s0008.xlsx Predicted AMR based on WGS data using BugSeq version 2025-06-02.

Table S8 jcm.00572-26-s0009.xlsx AMR phenotype of *P. aeruginosa* isolates in this study based on routine antibiotic susceptibility testing and interpretation using the CLSI guidelines, 34th ed (2024).

Table S9 jcm.00572-26-s0010.xlsx Degree of concordance between AMR genotype and AMR phenotype per isolate.

ASM does not own the copyrights to Supplemental Material that may be linked to, or accessed through, an article. The authors have granted ASM a non-exclusive, world-wide license to publish the Supplemental Material files. Please contact the corresponding author directly for reuse.
