# Supplementary material for: Applicability of Nanopore-only whole-genome sequencing for Pseudomonas aeruginosa outbreak investigation in the ICU setting: a multicentric study
Source: J Clin Microbiol. 2026 Jul 27;64(8):e00572-26. doi: 10.1128/jcm.00572-26 (PMC13464046; doi:10.1128/jcm.00572-26)
Supplement: Supplementary file 1 [file jcm.00572-26-s0001.docx]

**Supplementary material**

**Tables**

**Table S1.** Overview of the number of samples, the duration of sequencing and the total yield (successfully called Gigabases) per Nanopore WGS run.

**Table S2.** Overview of the core genome allelic profile of all *P. aeruginosa* isolates in the multicentric study.

**Table S3.** Overview of SNVs in longitudinally collected *P. aeruginosa* isolates.

**Table S4.** Pairwise comparison of SNVs between isolates from different patients belonging to the same sequence type.

**Table S5.** Overview of genes encoding virulence factors and the predicted serotype amongst *P. aeruginosa* isolates collected in this study.

**Table S6.** Detailed overview of plasmids in this study.

**Table S7.** Predicted AMR based on WGS data using BugSeq version 2025-06-02.

**Table S8.** AMR phenotype of P. aeruginosa isolates in this study based on routine antibiotic susceptibility testing and interpretation using the CLSI guidelines, 34th edd (2024).

**Table S9.** Degree of concordance between AMR genotype and AMR phenotype per isolate.

**Figures**

**Fig. S1.** Bugseq outbreak analysis based on refMLST allelic distance of Illumina and Nanopore WGS data of 19 *P. aeruginosa* ICU strains. The refMLST distance is indicated on the lines interconnecting the nodes which represent a single genome. Sequence types (ST) are highlighted in color shades. Nodes that are separated less than 20 alleles apart are assigned the same color.
